# Evidence for Alteration of Gene Regulatory Networks through MicroRNAs of the HIV-Infected Brain: Novel Analysis of Retrospective Cases

**DOI:** 10.1371/journal.pone.0010337

**Published:** 2010-04-26

**Authors:** Erick T. Tatro, Erick R. Scott, Timothy B. Nguyen, Shahid Salaria, Sugato Banerjee, David J. Moore, Eliezer Masliah, Cristian L. Achim, Ian P. Everall

**Affiliations:** 1 Department of Psychiatry, University of California San Diego, La Jolla, California, United States of America; 2 HIV Neurobehavioral Research Center, University of California San Diego, San Diego, California, United States of America; 3 Department of Neurosciences, University of California San Diego, La Jolla, California, United States of America; 4 Department of Psychiatry, University of Melbourne, Melbourne, Victoria, Australia; Ohio State University Medical Center, United States of America

## Abstract

HIV infection disturbs the central nervous system (CNS) through inflammation and glial activation. Evidence suggests roles for microRNA (miRNA) in host defense and neuronal homeostasis, though little is known about miRNAs' role in HIV CNS infection. MiRNAs are non-coding RNAs that regulate gene translation through post-transcriptional mechanisms. Messenger-RNA profiling alone is insufficient to elucidate the dynamic dance of molecular expression of the genome. We sought to clarify RNA alterations in the frontal cortex (FC) of HIV-infected individuals and those concurrently infected and diagnosed with major depressive disorder (MDD). This report is the first published study of large-scale miRNA profiling from human HIV-infected FC. The goals of this study were to: 1. Identify changes in miRNA expression that occurred in the frontal cortex (FC) of HIV individuals, 2. Determine whether miRNA expression profiles of the FC could differentiate HIV from HIV/MDD, and 3. Adapt a method to meaningfully integrate gene expression data and miRNA expression data in clinical samples. We isolated RNA from the FC (n = 3) of three separate groups (uninfected controls, HIV, and HIV/MDD) and then pooled the RNA within each group for use in large-scale miRNA profiling. RNA from HIV and HIV/MDD patients (n = 4 per group) were also used for non-pooled mRNA analysis on Affymetrix U133 Plus 2.0 arrays. We then utilized a method for integrating the two datasets in a Target Bias Analysis. We found miRNAs of three types: A) Those with many dysregulated mRNA targets of less stringent statistical significance, B) Fewer dysregulated target-genes of highly stringent statistical significance, and C) unclear bias. In HIV/MDD, more miRNAs were downregulated than in HIV alone. Specific miRNA families at targeted chromosomal loci were dysregulated. The dysregulated miRNAs clustered on Chromosomes 14, 17, 19, and X. A small subset of dysregulated genes had many 3′ untranslated region (3′UTR) target-sites for dysregulated miRNAs. We provide evidence that certain miRNAs serve as key elements in gene regulatory networks in HIV-infected FC and may be implicated in neurobehavioral disorder. Finally, our data indicates that some genes may serve as hubs of miRNA activity.

## Introduction

RNA may be the most ancient form of biological phenomena, with functions stretching from templating DNA [1] to enzymatic self-regulation [2]. An intriguingly small, but functionally robust, RNA species known as microRNA has proved to be at the center of regulating genomic expression.

### MicroRNAs

**Figure 1:**
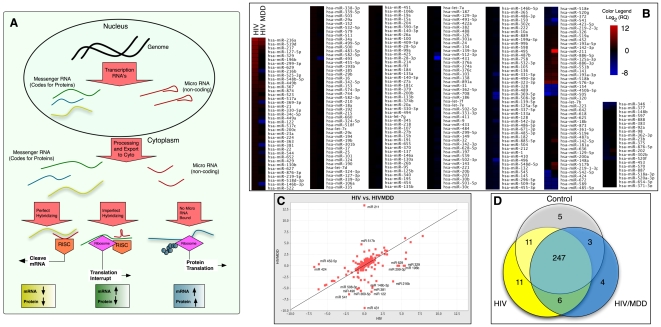
MicroRNA Profile of Frontal Cortex in HIV and HIV/MDD. (A) Summary of the biology of miRNAs. MiRNAs are synthesized in the nucleus and transported to the cytoplasm where they undergo processing by DROSHA and Dicer to mature miRNAs, which are presented by the RISC complex to hybridize to target sequences in the 3′UTR of mRNAs, where either translation is interrupted or mRNA is degraded. (B) Heat Map of miRNA Expression. While many miRNAs are not altered, a subset is upregulated and a smaller set downregulated in HIV. (C) MiRNA Expression of HIV vs HIV/MDD. Those whose expressions values fall on the x = y line would be contributed by HIV and those off of the line would presumably be related to MDD, more miRNA expression appears suppressed in the MDD context compared to HIV alone. (D) Venn Diagram of miRNAs expressed in the various groups. Supplementary Files S1 and S2 were used to generate (A) and (B) and can be manipulated using Gene Pattern software.

MiRNAs are short strands of 18-25 nucleotides that are both evolutionarily conserved and emergent, potentially currently undergoing evolution in vertebrates [3]. MiRNAs can be transcribed from non-protein-coding genomic regions or intronic regions of “host” genes, with which they are usually co-expressed [4]. Pre-miRNAs are synthesized in the nucleus, possessing characteristic hairpin loops [5]. After being exported to the cytoplasm they are further processed by Dicer enzyme into mature miRNAs usually of 21–23 nucleotides in length [6]. They function in association with the RNA-induced silencing complex (RISC) to hybridize to 8-mer “seed” sequences in the 3′ untranslated regions (3′UTR) of target mRNAs [7]. Imperfect matching results in interruption of translation by the ribosome and perfect seed-sequence matching leads to cleavage of the target mRNA [8]. The search for miRNA targets has been largely bioinformatics-based (the Sanger database is an excellent archive of information on pre-, mature- miRNAs, host genes, chromosomal location, and targets [9]). A schematic representation of the biology of miRNA is illustrated in Figure 1a.

#### MicroRNAs in Host Defense

Several recently published reports have demonstrated a complex interplay of viral products and host miRNA mechanisms. A novel and notable example of miRNA and virus interaction is the co-opting of miR-122 for replication by the hepatitis C virus, possibly explaining the predilection of hepatitis C for the liver. A microRNA link to viral tropism has also been suggested for HIV by Xu Wang and colleagues with their finding that specific miRNAs in monocytes may confer resistance to HIV infection in monocytes and loss of these miRNAs in macrophages increases susceptibility to infection [10]. MiRNA abundances can also change in response to various viral infections. A miRNA profile of peripheral blood mononuclear cells in HIV-infected individuals demonstrated that at least 62 miRNAs are altered in HIV infection [11]. A string of papers has even suggested that HIV can have a generalized suppressive effect upon the miRNA biogenesis pathway, possibly through inhibition of DICER [12], [13], however this finding is controversial.

#### MicroRNAs in the Central Nervous System

A growing body of evidence supports a role for miRNAs in neural patterning, neuronal maintenance and neurodegeneration. Kapsimali et al found that miRNAs have variable expression profiles in the developing and mature brain, demonstrating differences across the lifespan of a cell [14]. MiR-92b varies in expression during the transition from proliferation to differentiation of neurons, while miR-124 is constitutively expressed in mature neurons. Lau and colleagues identified 43 miRNAs with dynamic expression during transition from progenitor cells to mylenating mature oligodendrocytes miRNAs [15]. Unsurprisingly, miRNAs can be regionally specific, as evidenced by miR-222 which is restricted to the telecephalon [14]. MiRNAs can also be cell-type specific, e.g. the motor-neuron specific miR-218 [14].

MiRNAs have been implicated in neurodegenerative diseases such as: Alzheimer's, Parkinson's, and Huntington's chorea. MiRNA expression profiling of CNS tissue from patients with sporadic Alzheimer's disease revealed 13 dysregulated miRNAs, which included those with putative binding sites in the 3′UTR of amyloid precursor protein [16]. Kim and coworkers described a regulatory feedback network in midbrain dopaminergic cells whereby miR133-b and the gene Pitx3 regulate tyrosine hydroxylase function, possibly contributing to Parkinson's pathophysiology [17]. In the FC of Huntington's Disease patients, miR-132 was found to be significantly downregulated possibly resulting in increased levels of RE1-silencing transcription factor (REST), a protein with potential implications in neuropathogenesis [18].

#### MicroRNAs in NeuroAIDS

Given that miRNAs play a role in development and homeostasis of the CNS, and also are affected by and affect the properties of viral infection, it is important to clarify the interplay between miRNAs and HIV in the CNS and how they correlate with common clinical co-morbidities such as MDD. Interestingly, Eletto and colleagues found that HIV-Tat may promote miR-128a activity, leading to a reduction in SNAP25 expression in neurons [19], [20]. Neurologic dysfunction is likely influenced by overlapping mechanisms of miRNA dysregulation in neurodegenerative disorders, psychiatric disorders, and/or infectious diseases.

In the post-genome era, large-scale gene expression arrays have been employed to uncover biological processes of cellular function, tissue development, and biomedical pathologies. Message RNA profiling has been useful in identifying gene networks that coalesce into functional outcomes. However, gene expression arrays are informative for only one level in the central dogma of molecular biology: transcription from DNA to RNA. Translation of RNA to protein, involves regulatory factors that also affect downstream molecular outcomes. This report presents a snapshot of two levels of molecular biology (microRNA and messageRNA) in adult human frontal cortex (FC) autopsy tissue.

We present the first published large-scale profiling of miRNAs in the frontal cortex of HIV and HIV/MDD individuals. Our three goals were to: identify changes in the miRNA profile in the CNS associated with HIV-infection, determine whether a clinical diagnosis of MDD in the setting of HIV-infection alters miRNA profiles, and integrate miRNA expression with gene expression data in order to identify correlations of miRNAs with their putative targets in the CNS.

## Results

### Baseline Characteristics

RNA was isolated from 3–4 individuals of three selected groups: control, HIV, and HIV/MDD. We attempted to match subjects on several key variables (post-mortem interval, sex, race, age, MDD episode within 6 months, and HIV viral load,). No subjects met criteria for substance abuse or dependence. Table 1 displays the demographic data for each subject. By attempting to closely-match our subjects within and between groups it was our hope that any observed differences in miRNA abundance were due to viral infection and/or psychiatric diagnosis.

**Table 1:** Characteristics of Cases in miRNA Study.

Group	Study ID	Pathology	MDD	HIVE	Age	PMI	Sex
*Control*	1	Normal	None	n/a	50	11	M
*Control*	2	Normal	None	n/a	24	17	M
*Control*	3	Normal	None	n/a	34	9	M
*HIV*	CC103	Min. Non-Diganostic abnormalities	None	No	54	12	M
*HIV*	CC141	Bacterial leptomeningitis	None	No	56	12	M
*HIV*	CC163	Normal	None	No	45	12	M
*HIV/MDD*	CA110	Encephalitis	6 Mo	Yes	43	12	M
*HIV/MDD*	CE111	Other Non-Infect	Current	No	34	10	M
*HIV/MDD*	CE143	Normal	6 Mo	No	38	7	M

Abbreviations: MDD—Major depressive disorder, 6 Mo–within six months, PMI–post-mortem interval to autopsy, HIVE–neuropathologically confirmed HIV-encephalitis.

### Quality Control and Platform Validation Results

In order to identify semi-quantitative differences in miRNA abundance, several quality control experiments were performed to ensure accurate amplification and rational normalization. A ‘Xeno-RNA’ assay (TaqMan® Cells-to-CT™ Control Kit, Applied Biosystems) was performed, in which non-human, unrelated RNA was spiked into varying dilutions of human frontal cortex RNA stocks (pooled and non-pooled), keeping final reaction volumes constant. A standard RT-PCR program was run and the amplification plots were inspected to detect the presence of PCR-inhibitors or RNAses within the isolated RNA stocks. The amplification plots did not demonstrate any evidence of PCR-inhibition or RNAse activity (see Supplementary Figure S1). Furthermore, high-concentration polyacrylamide electrophoresis of the human frontal cortex RNA failed to reveal evidence of non-specific degradation (Supplementary Figure S1).

The selection of a stably expressed normalization microRNA was also important in order to ensure accurate comparison between groups. In examining the amplification plots, (Supplementary Figure S2) we determined the optimal endogenous control to be RNU44 (Accession Number NR_002750), a small ribosomal RNA, which exhibited less sample to sample and group to group variability. The default endogenous control for the array platform (MammU6, Accession Number NR_004394, supplied in quadruplicate wells on the array) exhibited lower abundance in HIV/MDD pooled sample. When the single subject RNA samples were subsequently analyzed, this effect was confirmed (Supplementary Figure S2). The etiology of the reduced abundance of MammU6 in HIV/MDD FC samples along with the increased observed variability in abundance between groups remains unclear.

To perform large-scale miRNA profiling we employed a pooled-RNA technique. Whereby equal masses of total RNA from a number of subjects in a group classification (Control, HIV, HIV/MDD) were physically pooled and subjected to quantitative PCR, a procedure that has been analyzed and modeled for microarray experiments in Peng et al [21]. Peng et al illustrated that pooling RNA samples was a cost-effective tool for discovery science which yielded results representative of group by group differences, as long as the groups are sufficiently different and defined [21]. In order to assess the efficacy of this procedure, we compared individual Taqman qPCR assay results from wells using single subject RNA to wells using pooled RNA of those same individual subjects. All analytical and mathematical procedures utilized in the Taqman miRNA array experiments described below were kept constant for these quality control experiments.

We verified that pooled miRNA expression results reproduced the average of single subject values, though as modeled in Peng et al [21], this likely represents a group difference (Supplementary Figure S3). We chose miRNAs that were found to be upregulated, downregulated, and not changed in the HIV and HIV/MDD groups when compared to controls. Concordance between pooled and single subject results were demonstrated for 9 out of 10 miRNAs selected for analysis: miRs-134, -154, -132, -122, -214, -29a, -495, -193a, -125a. MiR-367, however, displayed discordant results. The pooled RNA samples showed increased expression of mir-367 in the HIV and HIV/MDD groups, while one-half of the single subject samples from the control and HIV/MDD groups failed to amplify. Although the groups differed on average, this was not statistically significant (Supplementary Figure S3). For quality control purposes, we isolated RNA from the same tissue a second time to verify consistency of observed miRNA abundance. We were pleased to find reproducible results (Supplementary Figure S4).

#### Taqman MiRNA Array Profiles

The microfluidic miRNA array uses a PCR-based platform, and experiments were performed in technical triplicate. We found significant differential expression of numerous miRNAs in HIV-infected frontal cortex. When comparing the HIV group with Control, 59 miRNAs were up- or down-regulated by at least twofold, and in the HIV/MDD group 78 miRNAs met these criteria. Figure 1b shows the heatmap of the expression profiles of HIV and HIV/MDD compared to Control (Supplementary Files S1 and S2 are available for analysis and manipulation using Gene Pattern [22]). Data files of these experiments are available on the Gene Expression Omnibus (GEO) database under the accession number GSE17491.

Each group possessed a unique subset of miRNAs, amplifying in only that condition. The Venn diagram in Figure 1D shows that five miRNAs were present in Control, but absent in HIV conditions. A total of 20 miRNAs were present in the two HIV groups and absent in Control. However, 11 miRNAs were present in HIV and not in the MDD subjects (Figure 1D). In HIV/MDD, those miRNAs which were expressed in both HIV and MDD, generally showed lower expression compared to control. To illustrate how the HIV and HIV/MDD profiles differed, the expression values for each miRNA are displayed on a scatterplot with HIV/MDD on the y axis and HIV on the x axis (Figure 1C). Many miRNAs were unchanged, as indicated in the heatmap of Figure 1B) exhibited by the clustering of points about the origin in Figure 1C. Any miRNA whose expression fell along the x = y line would indicate the expression levels were the same or similar in both HIV and HIV/MDD and deviations from this line would presumably be contributed by the presence of major depressive disorder. Most of the points that deviated were below the line, indicating that the cellular conditions of MDD in the frontal cortex might involve suppression of miRNA expression.

#### Chromosomal Locations

**Figure 2:**
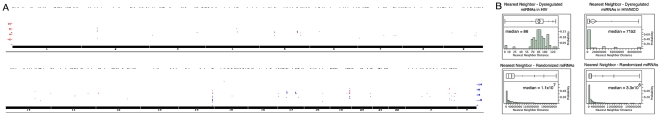
Chromosomal locations of dysregulated miRNAs. (A) Map of genomic locations of miRNAs dysregulated in HIV (red) and HIV/MDD (blue). (B) Distribution of nearest neighbor distances of dysregulated miRNAs in HIV (left) and HIV/MDD (right) and the distribution of nearest neighbor distances if randomly chosen miRNAs were calculated. The chromosomal locations were mapped in (A) using Supplementary Files S3, S4, S5 in the UCSC Genome Browser, for comparison, locations of all human miRNAs are shown in black. For each miRNA dysregulated, the distance to the nearest dysregulated neighbor was calculated in basepairs; and the distribution of distances was plotted in (B) along with the distribution calculated if miRNAs were randomly chosen from all known human miRNAs and nearest miRNA were calculated.

We next sought to determine whether there was a pattern with respect to chromosomal location of the dysregulated miRNAs. MiRNAs are distributed throughout the genome with the exception of the Y chromosome. In order to determine whether the dysregulated miRNAs were chromosomally clustered near one another, we mapped the location of the miRNAs dysregulated at least twofold (in either direction) along the human genome using UC Santa Cruz Genome Browser shown in Figure 2a [23]. The red points indicate miRNAs dysregulated in HIV and in blue, HIV/MDD. The y-axis indicates the Log_2_(RQ), and the x-axis indicates chromosome number and location. A supplementary file is available for browsing on the UCSC Genome Browser (Supplementary File S3 [23]). As a basis for reference, all the human miRNAs are plotted in black as annotated by the Sanger Databases [9]. Dysregulated miRNAs did seem to disproportionately cluster at Chromosomes 14, 17, 19, and the X chromosome. Figure 2a indicates transcriptional suppression in HIV/MDD on Chromosomes 14 and X; and activation on Chromosome 17.

In order to formally demonstrate whether the dysregulated miRNAs clustered closer to one another than due to chance alone, we calculated how far apart they were on the genome compared to choosing random miRNAs from the list of all available as in Shalgi et al [24]. The chromosomal locations of the two-fold dysregulated miRNAs were mapped out and the distance to the nearest-dysregulated-neighbor of each one was calculated and we constructed a histogram showing the distribution of distances to nearest-neighbor-dysregulated miRNA is displayed in Figure 2b. In HIV/MDD, the degree of clustering was more pronounced than the degree of clustering in HIV alone. This could indicate specific chromosomal suppression in the MDD state that is not present in the HIV-alone state. Given that we assayed only mature miRNAs, our observation may also indicate alterations in miRNA processing of specific miRNA families.

#### MiRNAs and Gene Targets

From the same subject pool and brain region (frontal cortex), our group compared message RNA levels (Accession Number GSE17440) of HIV to HIV/MDD individuals. The goal was to identify dysregulated mRNA expression signatures in order to differentiate HIV-positive subjects suffering from MDD from those who were did not have a diagnosis of MDD. These subjects are described in Table 2. Utilizing this mRNA data, we performed a target bias analysis as described in Methodsin order to determine the relationship between miRNA dysregulation and target-gene dysregulation. Table S1 shows the ANCOVA results of the 392 mRNA transcripts that were dysregulated in either direction with a p-value less than 0.05. These genes are each potential targets of miRNAs. Table S2 lists all miRNAs dysregulated ≥2-fold in either direction along with any corresponding target appearing in the ANCOVA analysis; the table is organized by miRNA and with genes listed in ascending order of statistical significance. In order to determine whether a dysregulated miRNA has a significant proportion of targets dysregulated, we used the hypergeometric probability distribution to calculate p-values to answer the question, “Given that a particular miRNA has m number of potential targets that appear on the gene array, which has a total of N unique genes which were detected in the FC, n of which were dysregulated at p<0.05, what is the probability that at least k targets of the particular miRNA would be dysregulated?” SPSS was used to calculate these probabilities for every dysregulated miRNA. In the gene array, we calculated the exact p-values from one-way ANCOVA. Since p<0.05 is arbitrary [25], we can calculate a target-bias probability for any other gene-array significance window, we did this for significance windows of p<0.01, p<0.005, and p<0.001 (which had 70, 39, and 6 genes, dysregulated respectively; shown in Table S2).

**Table 2:** Characteristics of Cases in Affymetrix Microarray Study.

Group	Study ID	Pathology	MDD	HIVE	Age	PMI	Sex
*HIV*	CC103	Min. Non-Diganostic abnormalities	None	No	54	12	M
*HIV*	CC141	Bacterial leptomeningitis	None	No	56	12	M
*HIV*	CC147	Normal	None	No	50	15	M
*HIV*	CA146	Normal	None	None	47	12	M
*HIV/MDD*	CA110	Encephalitis	6 Mo	Yes	43	12	M
*HIV/MDD*	CA143	Normal	12 Mo	No	38	7	M
*HIV/MDD*	CC207	Normal	12 Mo	No	56	12	M
*HIV/MDD*	CA236	Encephalitis	6 Mo	Yes	34	4	M

Abbreviations: MDD—Major depressive disorder, 6 Mo–within six months, 12 Mo–within 12 months, PMI–post-mortem interval to autopsy, HIVE – neuropathologically confirmed HIV-encephalitis.

**Figure 3:**
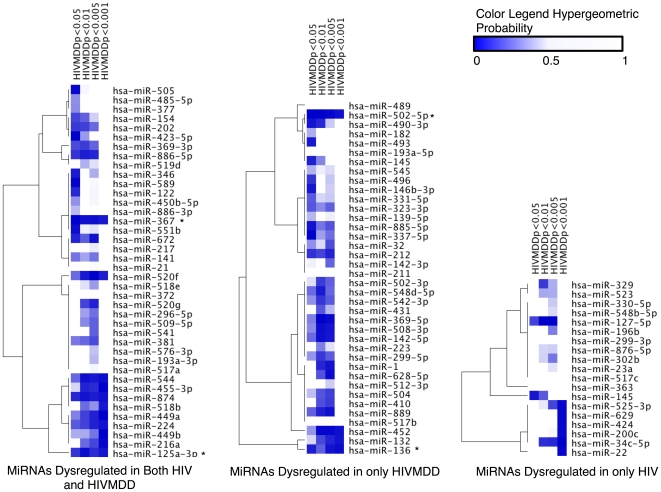
Target Bias analysis of a subset of miRNAs from frontal cortex in HIV and HIV/MDD conditions. MiRNAs dysregulated at least twofold in HIV or HIV/MDD are analyzed. Four windows of analysis are depicted: mRNAs dysregulated in HIV or HIV/MDD at p<0.05, p<0.01, p<0.005, and p<0.001. For each window, a list of dysregulated mRNAs is generated (n) from the Affymetrix U133 Plus 2.0 array (containing probes for N unique genes) which calculated relative quantification (RQ) of gene expression in Condition vs Control, data shown in Table S2. For each miRNA, a list of all targets present on the array is generated (m); from that list of targets, the number k targets are found to be significantly dysregulated within each of the four windows. Using cumulative hypergeometric distribution from SPSS software [41], we calculated the probability that k targets would be dysregulated from a list of size n genes, given that there are m targets on the gene array of the population N genes. (Using the function: P = 1-cdst.hypergeo(k,n,N,m) in SPSS). The color scale depicts the probability for target bias for each of 97 miRNAs and corresponding windows, with dark blue to white indicating 0.0<P<0.5. The miRNAs were sorted by their target biases using Hierarchical Clustering algorithm of Genepattern software. The gene expression in HIV/MDD of genes which are targets of miRNAs that show significant target bias is illustrated in Figure 4.

The target bias analysis is illustrated in Figure 3. The probabilities for each window and each miRNA are visualized in blue-shading scale indicating from lower to higher (0<p<0.5) and white indicating 0.5<p<1. The miRNAs seemed to separate into four general types. First, those which correlate to a larger number of miRNAs of lower significance value, indicated by dark blue in the p<0.05 column, for example miR-122 (Accession Number 406906), miR-589 (Accession Number 693174), and miR-423-5p (Accession Number 494335). Second, those miRNAs which had fewer gene-targets correlating to dysregulated mRNAs, but of higher significance, for example miR-518b (Accession Number 574474), miR-424 (Accession Number 494336), miR-629 (Accession Number 693214), miR-22 (Accession Number 407004), and miR-200b (Accession Number 406984) are indicated by the dark blue in the p<0.001 column. Third, those miRNAs which exhibited both patterns, namely many dysregulated gene targets and of high significance, indicated by blue across the rows. And finally, there were those that did not have a significant number of dysregulated targets, indicated by white across the row.

**Figure 4:**
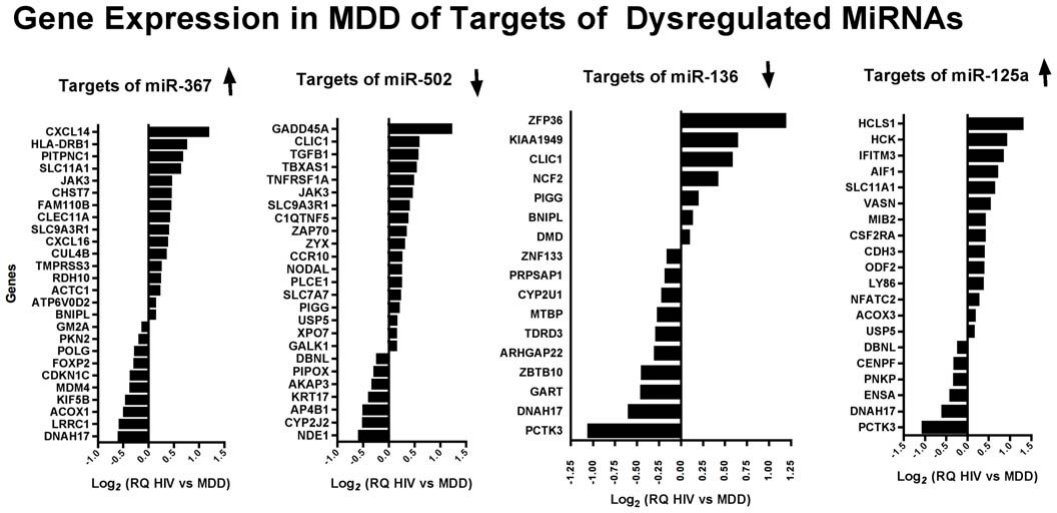
Targets of dysregulated miRNAs in MDD patients (mRNA relative quantification values). The gene expression values were determined using Affymetrix U133 Plus 2.0 array and compared HIV/MDD vs HIV alone from frontal cortex tissue from 6 patients in each group; one-way ANCOVA compared the two groups and were different at p<0.05 significance are listed in Table S1. The gene expression is plotted for those with 3′UTR seed sequences for miRNAs that showed highly significant target bias. The direction of the dysregulation of the miRNA is indication by the arrow; for miR-367, miR-136, and miR-125a, the majority of targets are dysregulated in accordance with the miRNAs; while for miR-502, the majority of targets are anticorrelated. ↑ or ↓ indicate the miRNA was upregulated or downregulated, respectively, in the array.

MiR-367 (Accession Number 442912), miR-125a-3p (Accession Number 406910), miR-502-5p (574504), and miR-136 (Accession Number 406927) showed significant target bias across all four windows (asterisks in Figure 3). The gene expressions (as determined compared to HIV-alone in the microarray) which are targets of these proteins are plotted in Figure 4.

**Figure 5:**
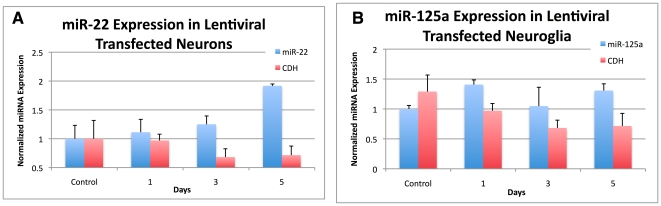
Lentiviral overexpression of (A) miR-22 and (B) miR-125a in primary neuronal cultures.

We sought to verify in vitro, two miRNA-target pairs. We used a lentiviral vector system to overexpress either a CDH-GFP control vector, miR-125a, or miR-22 under control of the CMV promoter. Vectors expressed the EGFP under the SV40 promoter. Figure 5 shows TaqMan qPCR indicating increased levels of miR-22 and miR-125a over time after exposure to the lentiviral vectors compared with the control vector.

**Figure 6:**
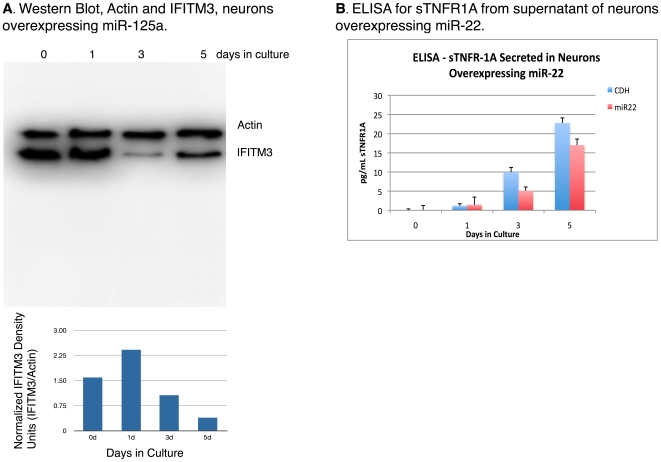
(A) Decreased protein production of IFITM3 in miR-125a overexpressing neuronal cells and (B) decreased secretion of sTNFR-1A in miR-22 overexpressing neuronal cultures.

Due to miR-125a's reported involvement in CNS development [26], [27], along with a reported dysregulation in the CSF of Alzheimer's disease [28], we investigated whether a given target of miR-125a would be affected by its overexpression. We found miR-125a to be upregulated in HIV and HIV/MDD, and 15/20 of its dysregulated target genes at the mRNA level to be upregulated (Figure 4). We tested whether protein levels of one of its targets, IFITM3, whose mRNA was also upregulated, would be affected by overexpression in primary neuronal cultures. We found that IFITM3 protein decreased in response to miR125a overexpression at 3-5 days of post-miR125a induction, Figure 6a. We have therefore provided in vitro evidence that miR-125a can alter protein levels of IFITM3 in primary human neuronal cultures.

We also sought to verify whether overexpression of miR-22 would affect protein levels of the predicted target sTNFR1A (soluble tumor necrosis factor receptor 1A). We chose to analyze sTNFR-1A because it is an important mediator of TNF signaling in the brain, it is suspected to play a role in a number of neurodegenerative diseases [29] and is implicated in inflammatory processes [30]. In our analysis, miR-22 showed significant target bias, meaning that a disproportionate number of its targets were dysregulated, including sTNFR-1A. Our in vitro study demonstrated that overexpressing miR-22 caused reduction in sTNFR-1A protein levels in the supernatant of primary human neurons.

## Discussion

### Limitations

Our study utilized a small sample size for a global gene-expression study, increasing probability for false-positives. For that reason, in our approach to the Target Bias Analysis, we analyzed for and presented thresholds for significantly dysregulated genes from a p<0.05 to also analyze for p<0.001, (Figure 3). Though we attempted to use patient samples from matching decades of life, given the resources available, we erred to minimize parameters that would lead to degradation of RNA such as post-mortem interval and brain pH. We chose a broad age-range in the control group to account for a younger average-age in the HIV/MDD group compared to the HIV-negative group (this is reflective of the available tissue at our resource). Our control group age range was 24–50, our HIV was 45–56, and our HIV/MDD was 34–43; we cannot rule out that our observations may be due to age group differences.

### Methodology and Platform

The miRNA array platform utilized in these experiments differs significantly from hybridization gene microarrays with respect to the chemical and physical dynamics. MiRNAs are highly conserved and cluster into families that are evolutionarily related and share sequence similarity. Further, by their nature of being relatively short strands, and in cells, they hybridize to many transcripts, increasing the potential for non-specific signals in a hybridization-based platform, such as microarray, is high. The PCR-based array employed here used a stem-looped multiplexed primer set for cDNA synthesis and begins from the 5′ end of the small RNA, which are unique to the mature miRNA. This feature precludes cDNA synthesis of other RNAs (even within the same family), mRNA or otherwise, that exist with sequence similarity. Our analysis was therefore specific for determining expression of mature miRNA sequences present in the tissue, which we believe to be functionally active.

### Snapshot of Two Levels of Molecular Function in Vivo

**Figure 7:**
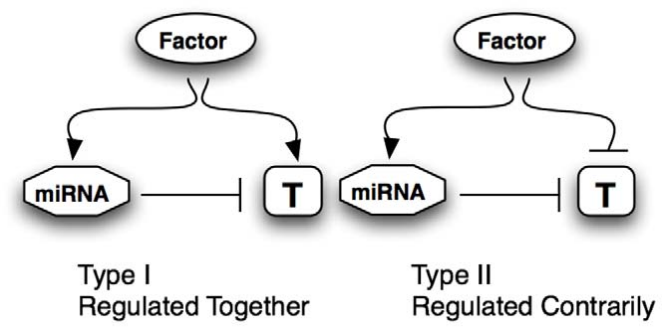
Two types of miRNA circuits in gene regulatory networks. In Type I, targets (T) are regulated together with the miRNA; or changes in expression would be correlated. In Type II, the upstream factor regulates the target and the miRNA contrarily, changes in expression would be anticorrelated. Reprinted from Molecular Cell, 26∶5, Tsang, Zhu, and van Oudenaarden, “MicroRNA-Mediated Feedback and Feedforward Loops are Recurrent Network Motifs in Mammals,” Copyright (2007), with permission from Elsevier.

As outlined in the model in Figure 1a, it is thought that perfect sequence homology leads to degradation of the target mRNA, while imperfect sequence homology results in interruption of translation [8]. Tsang, Zhu, and Oudenaarden [31] outlined two types of miRNA circuits, as shown in Figure 7. Type I circuits: miRNAs and their corresponding targets are regulated together, implying short-term modulation of protein translation and a regulation of homeostasis of gene function. Type II circuits: where miRNA circuits are inversely-regulated to corresponding targets; which we hypothesize would be circuits involved in long-term cellular phenotype, cell-lineage, or cell-differentiation functions. The majority of dysregulated genes in our dataset were dysregulated in the same direction as their target miRNA, implying more Type I regulatory circuits in the adult brain tissue.

More Type II circuits have been characterized than Type I circuits, as they are more readily identified and experimentally verified, Figure 7 [31]. We posit that these circuits are involved in tissue specificity and maintenance of cell identity, and would be regulated together with transcription factors. Type I circuits, in which the miRNA and its target are regulated together would allow for modulation of protein translation, maintenance of cellular homeostasis, or modulation of changes in local protein concentrations. For the purposes of neuronal functions in the CNS, protein translation (i.e. at the synapse) is a tightly regulated process for which miRNAs may play a crucial role [32]. Our observations here indicate evidence for many Type I circuits in the brain. This would provide a role for miRNAs regulating gene functions at the level of protein translation. This is a possible confounder when analyzing microarray data. Also, a protein may be translated or activated without any apparent change in gene transcript levels; and miRNAs are one of many mechanisms by which this could occur. Further, others have recently verified significant bias toward miRNA-mRNA-targets pairs being positively correlated using a empirical Bayes approach in Alzheimer's disease brain [33]. Together, this supports a hypothesis for Type I circuitry involvement in homeostasis maintenance and Type II circuitry involvement in tissue growth and differentiation.

MiRNAs may represent a common mechanism of cellular dysfunction leading to diverse neurobehavioral deficits. One miRNA, miR219, was shown to modulate NMDA-receptor-mediated neurobehavioral dysfunction and is implicated in the symptology of schizophrenia [34]. We found this miRNA to be upregulated in HIV/MDD, Figure 1b. A miRNA profile of Alzheimer's Disease patients showed significant overlap with our findings; 11/19 miRNAs found to be dysregulated in the FC in AD [28] were dysregulated in our study in the same direction, including miR125a, miR132, which have CNS developmental roles [14], and were highly significant in our target bias analysis (Figure 3).

The expression values from all miRNA targets are listed in Table S2. Recently, the gene pathway database and networking engine, Ingenuity Pathway Analysis [35], has added miRNAs to its repertoire. With the goal of identifying pertinent biochemical pathways, cell functions, or physiologic parameters, we analyzed these miRNAs which served as significant circuits in our target bias analysis; choosing those miRNAs which showed significant target bias, described above.

### MRNAs as Hubs of miRNA Activity

**Figure 8:**
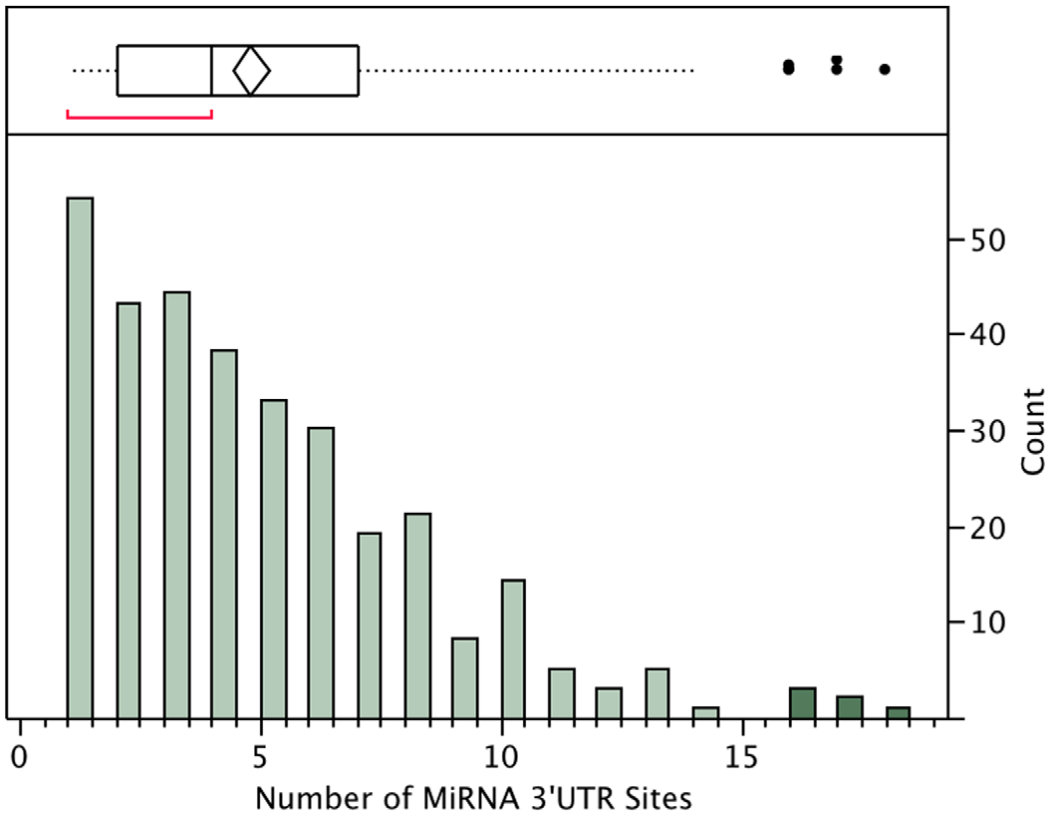
Number of dysregulated miRNA 3′UTR sites in dysregulated mRNAs. The number of miRNA targets sites for each dysregulated gene is counted and distribution shown (bottom) with outlier plot (top), six genes were outliers with 16–18 targets sites while the median was four target sites for each mRNA.

Until now, our analysis centered on miRNAs as circuits in gene regulatory networks, but genes themselves may have target sites for multiple miRNAs. In Table S2, some genes appear multiple times as targets of different miRNAs. Shalgi et al [24] identified genes as “hubs” of miRNA regulation that were under the post-transcriptional regulation of dozens of miRNAs. In plotting the distribution of the number of dysregulated miRNA sites in the dysregulated genes (Figure 8), we found the majority of our dysregulated genes had 3′UTR target sequences for few miRNAs (median of 4, and interquartile range 2–7), but a minority were determined to be extreme values in the distribution plot of Figure 8 with 16–18 miRNA target sites of dysregulated miRNAs. These genes are: cullin 4B (CUL4B, 8450), forkhead box P2 (FOXP2, 114142) regulator of g-protein signaling 10 (RGS10, 54290), chloride intracellular channel 1 (CLIC1, 1192), endosulfine α (ENSA, 2029), and colony stimulating factor receptor 2α (CSF2RA, 1438).

**Figure 9:**
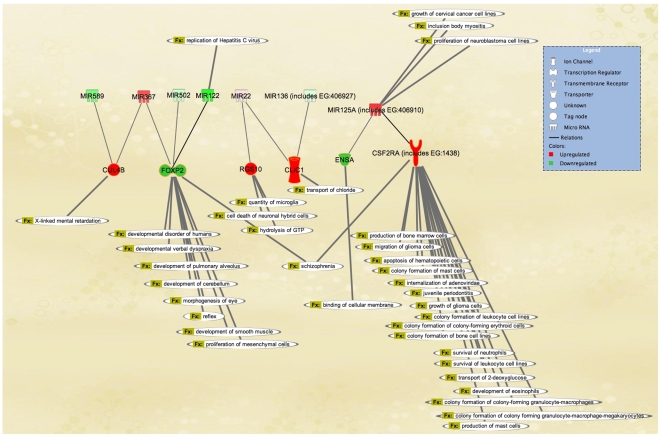
Pathway analysis of mRNAs and miRNAs. MiRNAs were chosen on the basis of their highly significant target bias, and mRNAs chosen that had a high number target sequence sites in 3′UTRs. Ingenuity Pathway Analysis algorithm, database, and software was used to illustrate the interrelationships and functional relationships.

Pathway analysis of this small list of genes using Ingenuity Systems' software revealed interesting functions in support of the hypothesis that they would be “hubs” of cellular or physiologic function and is illustrated in Figure 9 [35]. FOXP2 is a transcription factor important for some neuronal functions including reflex, development of cerebellum, developmental disorders, and is implicated in schizophrenia. RGS10 was upregulated and is functionally relevant to microglia quantity and cell death of neuronal hybrid cells. CUL4B was upregulated in MDD and forms a complex that functions as an E3 ubiquitin ligase that catalyzes polyubiquitination of specific protein substrates. CUL4B is required for proteolysis of regulators of DNA replication, and is thereby involved in cell cycle or DNA repair. CLIC1 is a nuclear chloride ion channel and regulates fundamental cellular processes including stabilization of membrane voltage potential, transepithelial transport, maintenance of intracellular pH, and is increased in our findings in FC of HIV patients with MDD compared to those without. Interestingly, others showed an increase after exposure to dexamethasone, an activator of glucocorticoid receptor [36]. ENSA was downregulated in MDD, and encodes the regulatory subunit of an ATP-dependent potassium channel which functions to inhibit potassium channel activity. CSF2RA, upregulated in MDD, is a transmembrane receptor, a member of the cytokine receptor family, and its signaling controls the production and differentiation of macrophages. According to Ingenuity, it is implicated in many diseases including schizophrenia and HIV infection [35].

In vitro, we verified an effect of two miRNAs on two targets. Both miR-125a and miR-22 were found to be upregulated in our analysis of HIV-infected individuals. The target genes of these miRNAs were also upregulated, indicating Type-I circuitry. We provide evidence that the induction of these dysregulated miRNAs impact the protein translation of predicted mRNA targets. Specifically, we chose an intracellular membrane protein, IFITM3 and a secreted protein involved in neuroinflammation, sTNFR1A. Like other studies, the miRNA-mRNA target pairs here were positively correlated, and we showed in vitro, a decrease in protein content. These results support a functional outcome of positively correlated miRNA-target transcription to be potentially allowing for a buffer-effect of protein translation for homeostatic maintenance. A logical extension to these experiments is to test in a high-throughput manner, the protein content of these targets under the different developmental and disease-states and to determine which upstream factors first postulated by Tsang [31] differentially regulate miRNA-target transcription.

### Clinical Relevance

Major depressive disorder (MDD) is a clinically defined entity with little understanding as to the underlying pathological substrate. Biologically, MDD is characterized by disruption of neurotransmitters, especially serotonin and noradrenaline, which are the main targets of antidepressants. We previously demonstrated significant reduction of glial cell number in the cingulate and dorsolateral prefrontal cortical regions [37], [38], while Evans et al., [39] reported dysregulation of the trophic factors fibroblast growth factor (*FGF*) *2* and *9* in the brains of individuals with MDD. We recapitulated the alteration in *FGF2* and *FGF9* in human brain aggregates exposed to cortisol [40]. There appears to be a disturbance of trophic factors and glial cell function in addition to neurotransmitter abnormalities contributing to MDD.

Unfortunately, the prevalence of MDD in the HIV population is still very high, despite a sharp fall in mortality rates due to effective antiretroviral treatment. It is possible that in this treatment era, living with chronic HIV infection may result in long-term neuropathological changes that predispose to MDD. For example, it is known that HIV is associated with a range of inflammatory pathologies, neuronal loss and dendrite-synaptic damage [41]. These neurodegenerative changes have been linked to neurocognitive impairment [42], [43], [44]; however, it is also possible that these changes potentiate MDD [38].

Large-scale gene expression analysis is daunting and the biological significance of the findings is difficult to ascertain. In a microarray analysis comparing gene expression in the CNS of HIV-infected individuals with neuropathologically confirmed HIV-encephalitis, our group reported changes in transcription of genes involved in signaling and the cytoskeleton (Accession Number GDS1726) [45]. Changes in the amount of a particular mRNA might not necessarily correlate to changes in function of protein for which it codes. Additionally, the function of a gene (i.e., protein translation) could be altered without concordant changes in transcript abundance. One mechanism by which these apparent digressions occur is through alteration of the miRNA repertoire in the tissue.

Currently, many gene-expression studies use microarray technology, the basis for which is RNA transcript hybridizing to immobilized probes which correspond to tens of thousands of defined sequences. The platform is analyzed by comparing the signal, through an enzymatic or fluorescence reaction, of a test-sample (ie: Disease patient) compared to some reference sample (ie: Control or healthy subject). In the current study, we augment information from microarray gene expression with analysis of miRNA profiling through a PCR-based array. Since the gene expression and miRNA expression analyses were performed from the same brain region, FC, on the same panel of patients, we are able to consider two levels of biology and identify MiRNAs as circuits in gene regulatory networks pertinent to molecular homeostasis in adult human FC. Future studies may seek to analyze other brain regions systemically using a similar methodology. We chose to begin with frontal cortex matter because it is the site of greatest damage in cortex by HIV and our group previously showed changes in the FC in MDD [38].

While the results we present are correlative in nature, and provide only a snapshot, it is quite telling that a viral infection; absent from other confounding factors such as drug dependence or neurocognitive impairment, impacts the miRNA repertoire of the FC. We also found that a subset of miRNAs were dysregulated, generally downregulated, in the FC of patients grouped by psychiatric diagnosis, (HIV/MDD compared to HIV alone). Cotter et al showed decreased synaptic and dendritic density in CNS of MDD patients [38]. Ashraf and Kunes hypothesized that miRNAs might serve as the molecular etiology for memory and presented evidence for RISC-mediated control of RNA stability underlying protein synaptic protein synthesis associated with memory [32], [46]. While it is accepted that neurons are not infected by the human immunodeficiency virus, we hypothesize that low-grade, chronic neuroinflammation, or neurovascular alterations owing to activated microglia or infiltrating monocytes would cause paracrine signaling to neurons leading to activation or suppression of RNA transcription, which would be consistent with our observation of miRNA-dysregulation being chromosomally clustered around certain regions of the genome. Interestingly, Manakov and others showed that miRNAs clustered on Chromosomes 14 and X were downregulated during neuronal development and synapse formation, concurring with our study in HIV and MDD [26].

### Conclusion

While other studies that integrated both miRNA and mRNA quantification focused on developmental and cellular biology, our study focused on a single time point in adult human brain tissue. For comparison, we compared healthy with infection and infection with a psychiatric disorder. MDD and other psychiatric disorders are characterized physiologically by pertubations in neuroendocrine system, the putative substrate that acts on the brain to give the psychiatric effect. We found a repertoire of miRNAs downregulated in MDD along with related gene-targets and gene “hubs” of miRNA activity. This may potentially serve as the molecular link between the neuroendocrine pertubations and the subtle pathologic changes that occur in MDD outlined above. The methodological platform for quantitation of miRNAs measured amplification of PCR reaction in solution rather than hybridization to immobilized complementary strands, it is highly sensitive and reproducible; and may even be more cost-effective than the traditional array platforms. Finally, our target bias analysis, which analyzed the results of a gene array yielding a host of dysregulated transcripts from the perspective of dysregulated MiRNAs–a potentially powerful analytic tool applicable to many tissues, diseases, developmental conditions, and species that will help elucidate the regulatory role of miRNAs in complex molecular networks.

## Materials and Methods

### Subject Acquisition

#### Ethical Statement

The University of California San Diego Institutional Review Board approved the work included in this project for continuing use of anonymous pathological specimens and clinical data. Written informed consent was obtained from all subjects included in the study.

#### Microarray

For the microarray study, we included eight HIV+ cases from the HIV Neurobehavioral Research Center at the University of California San Diego. Cases had neuromedical and neuropsychological examinations within a median of 6 months before death. Most cases died as a result of acute bronchopneumonia or septicemia, and autopsy was performed within 24 h of death. We identified four cases that had a lifetime diagnosis of MDD, two of whom also had HIV encephalitis. Table 2 summarizes the cases. At time of autopsy, tissue from the frontal lobe was flash frozen and stored indefinitely at −80°C.

#### MicroRNA Array

From the patient-pool, three MDD and non-MDD cases were chosen on the basis of closely matching age, PMI, and excluding a history of substance dependence or abuse, and having a major depressive episode within six months, as determined by meeting DSM-IV criteria by a trained psychiatrist. Because of limited resources and requirement to closely match cases, all cases were male. Matched control cases were HIV-, had no history of psychiatric disorder and causes of death were unrelated to brain injury.

### Microarray

To determine the differentially expressed genes between patients with HIV and HIV+MDD, patient cDNA was hybridized to the Affymetrix U133+2 chip (Affymetrix, USA), using standard methods suggested by the manufacturer. All data are MIAME compliant and the raw data were deposited in the Gene Expression Omnibus (GEO) Accession Number GSE17440. The resulting *.cel files were imported into the Partek Genomics Suite software (Partek Inc, St. Louis, MO, USA) [47] to analyze probe intensity and to determine the differential gene expression between the groups.

### MicroRNA Array

Ten mg tissue from cortical grey matter of FC was dissected and RNA was isolated following instructions of MirVana RNA isolation kit (Applied Biosystems). 900 ng RNA from each subject was pooled into their respective groups (for a total of 2700 ng RNA). The RNA was reverse-transcribed using a MicroRNA Multiplex Primer set and MicroRNA Reverse Transcription kit (Applied Biosystems), cDNA was stored at −20°C. Equivalent to 800 ng of the cDNA product was applied to TaqMan MicroRNA Array v2 and reactions monitored and analyzed according to manufacturer's protocols in an Applied Biosystems 7900HT Fast Real-Time PCR System, applying the low density array settings and results interpreted in Applied Biosystems's RQ Manager software [48]. For relative quantification (RQ), the ∂∂CT method was used. The array supplied quadruplicate wells for mammalian U6 RNA as an endogenous control, along with RNU44 and RNU42; we determined RNU44 was the optimal endogenous control in our samples, and the RQ was calculated using the Control group as the calibrator. The arrays were performed in technical triplicates. These array data were deposited to the GEO database under the Accession Number GSE17440.

### Quantitative PCR of miRNA

Non-pooled and pooled RNA isolated above along with a second round of RNA isolation was reverse-transcribed and analyzed separately with TaqMan RT-PCR microRNA assays (Applied Biosystems). The reverse transcription is carried out with a specific stem-looped primer step for each individual miRNA followed by qPCR using FAM-labeled probes in 384-well optical plate in Applied Biosystems 7900HT Fast Real-Time PCR System. The following RNAs (Applied Biosystems product ID#s) were analyzed: MammU6 (#4395470), RNU44 (#4373384), miR-122 (#4395356), miR-29a (#4395223), miR-367 (#4373034), miR-142-5p (#4395359), miR-154 (#4373270), miR-214 (#4395417), miR-193a-3p (#4395361), miR-495 (#4381078), miR-125a-3p (#4395310), miR-134 (4373143). The assays were performed in technical triplicate and biological duplicate.

### In vitro Lentiviral Expression of miRNAs

Primary human neuronal cultures were maintained according to our past procedures [49]. Lentiviral vectors were obtained from Origene (Rockville, MD) which contained human miR-125a (#SC400749) and human miR-22 (#SC400286) mature sequences on a pCMV-MIR backbone. Neuronal cultures were exposed to 10 multiples of infection (MOI) 0, 1, 3, 5 days, RNA isolated and miRNA quantified by qPCR.

### Western Blotting and ELISA

Total protein cell lysates from cells treated exposed to miR-125a were obtained by incubation with a lysis buffer containing 0.15 M NaCl, 5 mM EDTA, 1% Triton X-100, 10 mM Tris-HCl, and 1% SDS with Complete Proteases Inhibitor Cocktail (Roche, Palo Alto, CA). Ten μg protein was loaded into 4-10% gradient polyacrylamide gel, separated by electrophoresis, and transferred to PVDF membrane. After blocking with 10% non-fat milk in PBS-0.3%Tween-20, membrane was probed overnight with rabbit anti IFITM3 (#10410, Abnova, Taipei, Taiwan) overnight at 4°C. Blots were visualized after incubating with secondary, peroxidase-conjugated anti-rabbit antibody (#PI-1000, Vector, Burlingame, CA) and incubated with chemiluminescence reagent (Pierce, Rockford, IL).

Two-hundred μL supernatant was removed at 1, 3, 5 days, and immediately following exposure to 10 MOI of miR-22 lentiviral vector. Media were replaced with neurobasal growth media following aliquot removal. In order to alleviate effect of differential evaporation, volume in each well was adjusted to 500 µL immediately prior to supernatant sample collection. Supernatant was stored in 10% bovine albumin-blocking solution to prevent analyte loss to labware. Samples were diluted 1∶2 and sTNFR1A was quantified using ELISA following manufacturer's protocols (kit DRT100, Quantikine, Minneapolis, MN).

### Data Analysis

The Taqman MicroRNA Array v 2 (Applied Biosystems) was performed in triplicate and yielded qPCR data analyzed in Applied Biosystems RQ Manager Software. Relative Quantification (RQ) was determined by the ∂∂CT method using RNU44 as an endogenous control transcript and the Control group signal as the calibrator. For illustration, we reported the Log_2_(RQ). Briefly: ∂CT_Sample_ = CT_RNU44_−CT_miRNA_; ∂∂CT_HIV_ = ∂CT_Control_−∂CT_HIV_; and RQ_HIV_ = 2^−∂∂CTHIV^ [50].

Since the samples for the microarray were not pooled, we could perform one-way analysis of covariance (ANCOVA) for covariates, age, PMI, MDD status, viral load, were inputted into the model. Robust Multichip Analysis (RMA) in Partek gene expression software was used to perform the ANCOVA to determine ratio by MDD-positive vs MDD negative and calculate p-values for each probe.

To determine the chromosomal locations of the miRNAs, the data (Chromosome number and basebair location) were extracted from Mirbase database, and the locations of the miRNAs which were dysregulated at least twofold in either direction was listed and mapped. The nearest neighbor of a dysregulated miRNA was calculated (only for neighbors on the same chromosome) to determine nearest neighbor distance for each dysregulated miRNA. Using JMP statistical software, a distribution plot was generated showing the distribution of the miRNA-nearest-neighbor distances for the dysregulated miRNAs to plot median and interquartile ranges. For comparison, a bootstrap randomization method was used. We randomly choose from a list of all miRNAs and calculated the nearest-neighbor distance if randomly chosen, this was performed 999 times and the distribution is shown.

A target bias analysis was performed for each dysregulated miRNA as in Tsang et al [22]. A list of targets of miRNAs for each dysregulated miRNA was generated from data extraction from Mirbase databse. Each miRNA had number, **m**, of unique genes that were both possible targets of a particular miRNA and present on the Affymetrix gene chip. The number, **N**, of genes expressed on the Affymetrix chip was determined. For a particular significance window (i.e.: p<0.05, p<0.01, p<0.005), a number, **n**, genes were dysregulated in the microarray. Each dysregulated miRNA also had a number, **k**, of possible targets that were also dysregulated at given signficance cutoff. The cumulative hypergeometric probability distribution (sampling without replacement) was used to calculate the probability that at least **k** targets of a particular miRNA would be on a list of size **n** dysregulated genes, given that **m** possible targets are on a chip of probeset size **N**. This probability was determined in SPSS [51] using the function: P = 1-cdst.hypergeo(**k**,**n**,**N**,**m**) and illustrated in color code.

In order to determine whether some genes that were dysregulated had a disproportionate amount of miRNA 3′UTR target sites which were also, dysregulated, we counted the number of 3′UTR sites in each and plotted the distribution using JMP software. There was a median of 4 sites for dysregulated miRNAs in the dysregulated genes, with four genes having up to 16–18 target sites being outliers as determined by JMP statistical software.

## Supporting Information

Figure S1 (A) XenoRNA assay. (B) Polyacrylamide electrophoresis and SybrGold staining of total RNA. Pooled RNA samples run on the array-cards were serial tenfold diluted and known quantity of xeno-added, ACTB and Xeno-RNA were measured by TaqMan reverse-transcription-PCR. Plotted are CT versus mass RNA, ACTB CT values increased proportionately with decreasing RNA and Xeno-RNA remained unchanged across dilutions; indicating no RNAses and consistent reverse transcription efficiency
(2.12 MB PDF)

Figure S2 Amplification plots for MammU6 and RNU44 from (A) the miRNA Array 2.0 and (B) individual qPCR reactions of subjects. (A) From the array, it was apparent that in HIV/MDD, MammU6 was differentially expressed compared to the other groups, which is confirmed (B) in individual, non-pooled, samples performed in biological duplicate.
(12.51 MB PDF)

Figure S3 Pooled and Non-pooled Expression by Group of Selected miRNA's Using qPCR. The pooled RNA samples that had been included in the Taqman miRNA Array v2.0 was subjected to reverse-transcriptase real-time PCR for primers/probes for miR134 (Accession Number 406924), miR154 (Accession Number 406946), miR132 (Accession Number 406921), miR122, miR214 (Accession Number 406996), miR29a (Accession Number 407021), miR495 (Accession Number 574453), miR193a (Accession Number 406968), miR125a, and miR367. The RNA from individual subjects that comprised the pooled samples were also subjected to RT-PCR and plotted together with the pooled sample. (Closed Box) Pooled Control, (Open Box) Control subjects, (Closed Triangle) Pooled HIV, (Open Triangle) HIV subjects, (Closed Circle) Pooled HIV/MDD, (Open Circle) HIV/MDD subjects. ↓ indicates no amplification for a subject.
(1.05 MB TIF)

Figure S4 Reproducibility - Effect of Tissue Dissection. RNA was isolated on two separate occasions following the miRVana protocol from the frontal cortex tissue and subjected to RT-PCR for miR122 (diamond), miR125a-3p (square), miR132 (triangle), and miR134 (X), and miR495 (asterisk). RNU44 was used as an endogenous control, and the Log2(RQ) is plotted (median and range of technical triplicate measurements); for calibrator, the median control subject was used. The signal from the RNA Extraction A vs RNA Extraction B is plotted; perfect reproducibility would be along the x = y line.
(0.51 MB PDF)

File S1 For analysis and visualization of heatmap with Genepattern software, used with File S2.
(0.02 MB TXT)

File S2 For analysis and visualization of heatmap with Genepattern software, used with File S1.
(0.01 MB TXT)

File S3 For analysis and visualization of chromosomal locations of miRs' genomic locations, for use with the UCSC Genome Browser, used with Files S4 and S5.
(0.01 MB TXT)

File S4 For analysis and visualization of chromosomal locations of miRs' genomic locations, for use with the UCSC Genome Browser, used with Files S3 and S5.
(0.00 MB TXT)

File S5 For analysis and visualization of chromosomal locations of miRs' genomic locations, for use with the UCSC Genome Browser, used with Files S3 and S4.
(0.00 MB TXT)

Table S1 One-Way ANCOVA - Affymetrix Array of Genes HIV vs HIV/MDD.
(0.05 MB TXT)

Table S2 MiRNA's which are twofold dysregulated and their dysregulated target-genes, sorted by significance.
(0.04 MB TXT)
