# Supplementary figures and images for: Evidence for Alteration of Gene Regulatory Networks through MicroRNAs of the HIV-Infected Brain: Novel Analysis of Retrospective Cases
Source: PLoS One. 2010 Apr 26;5(4):e10337. doi: 10.1371/journal.pone.0010337 (PMC2859933; doi:10.1371/journal.pone.0010337)

A

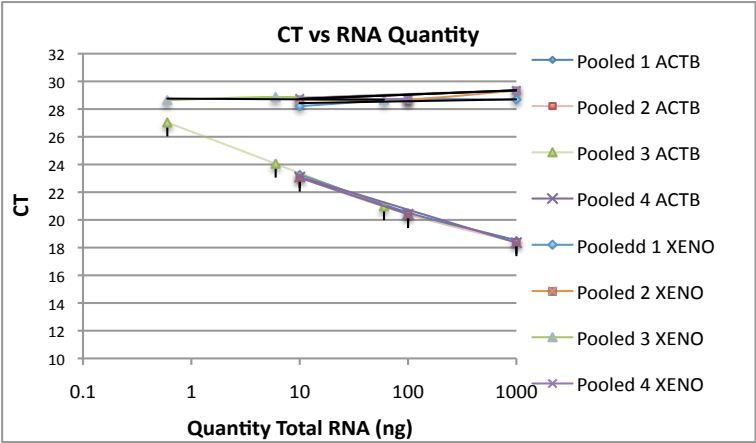

B

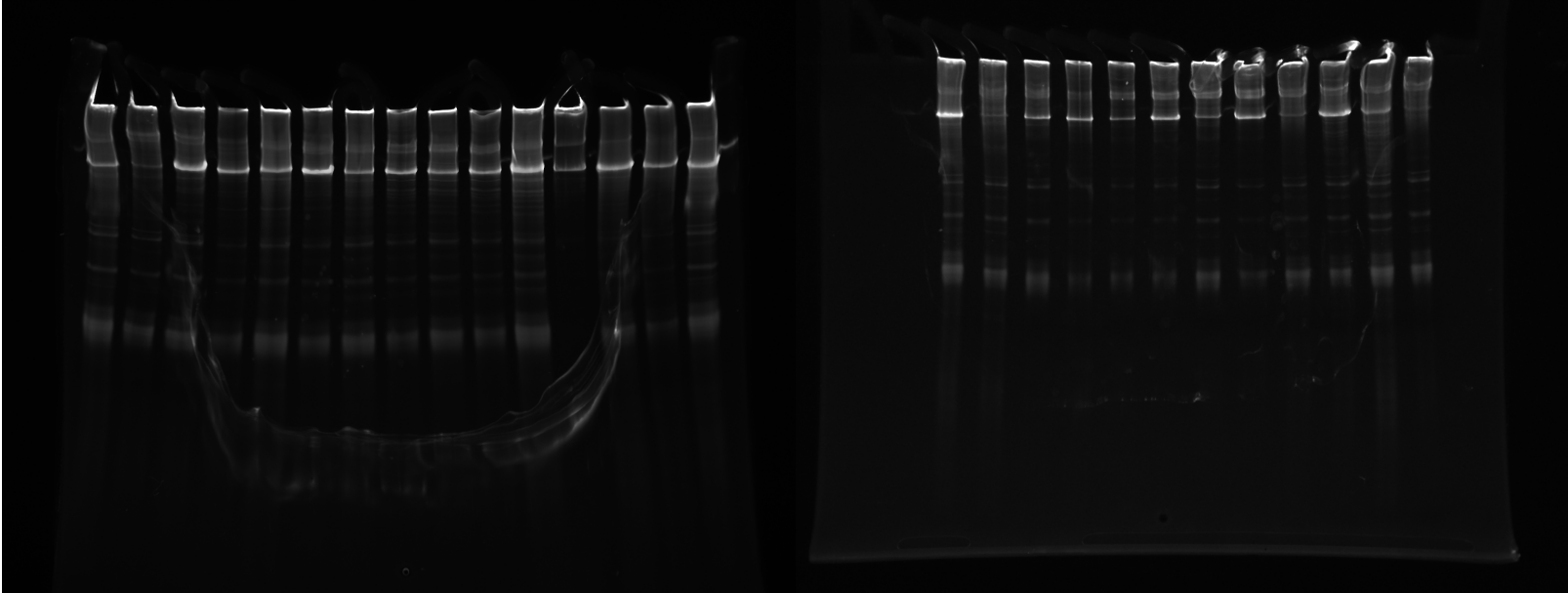

Supplement: Figure S1 — (A) XenoRNA assay. (B) Polyacrylamide electrophoresis and SybrGold staining of total RNA. Pooled RNA samples run on the array-cards were serial tenfold diluted and known quantity of xeno-added, ACTB and Xeno-RNA were measured by TaqMan reverse-transcription-PCR. Plotted are CT versus mass RNA, ACTB CT values increased proportionately with decreasing RNA and Xeno-RNA remained unchanged across dilutions; indicating no RNAses and consistent reverse transcription efficiency (2.12 MB PDF) [file pone.0010337.s001.pdf]

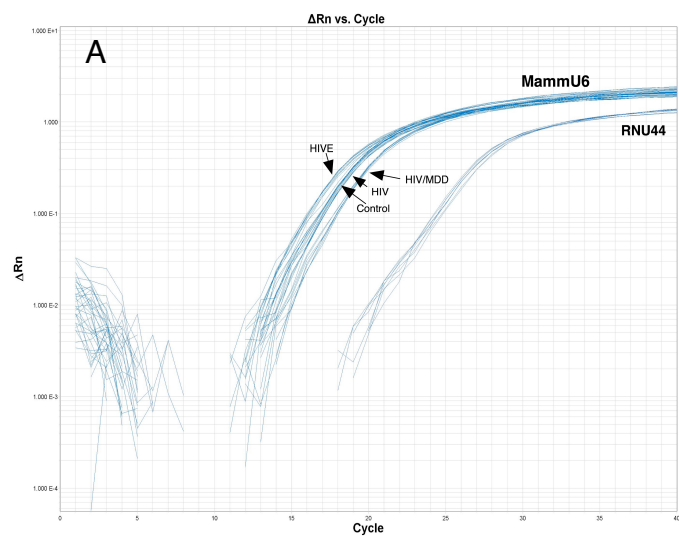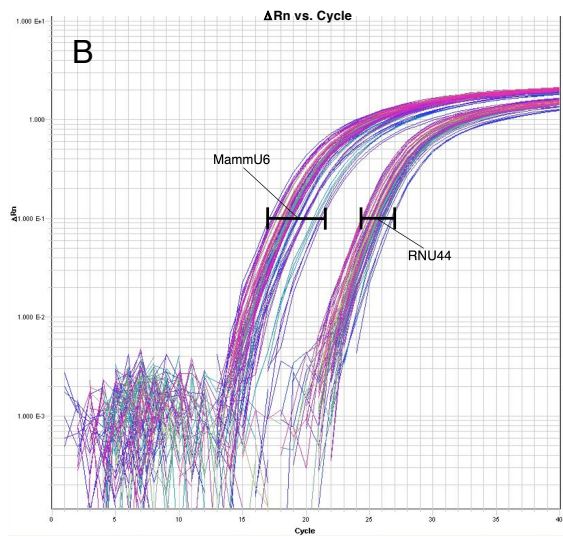

Supplement: Figure S2 — Amplification plots for MammU6 and RNU44 from (A) the miRNA Array 2.0 and (B) individual qPCR reactions of subjects. (A) From the array, it was apparent that in HIV/MDD, MammU6 was differentially expressed compared to the other groups, which is confirmed (B) in individual, non-pooled, samples performed in biological duplicate. (12.51 MB PDF) [file pone.0010337.s002.pdf]

## Reproducibility - Effect of Tissue Dissection

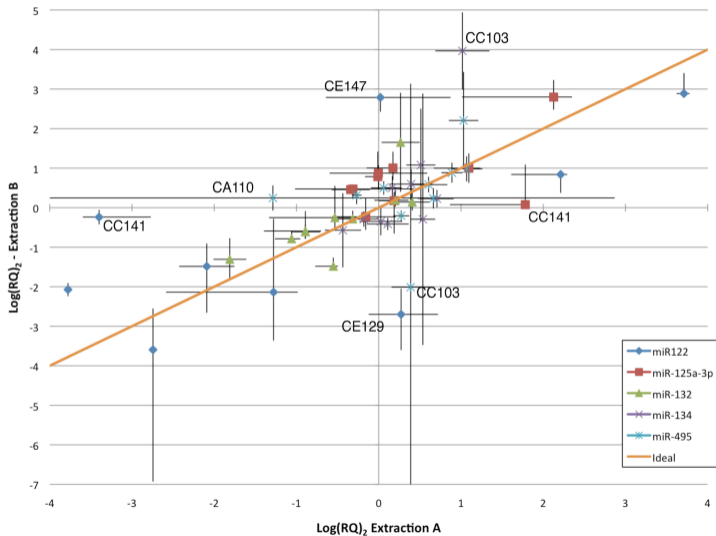

Supplement: Figure S4 — Reproducibility - Effect of Tissue Dissection. RNA was isolated on two separate occasions following the miRVana protocol from the frontal cortex tissue and subjected to RT-PCR for miR122 (diamond), miR125a-3p (square), miR132 (triangle), and miR134 (X), and miR495 (asterisk). RNU44 was used as an endogenous control, and the Log2(RQ) is plotted (median and range of technical triplicate measurements); for calibrator, the median control subject was used. The signal from the RNA Extraction A vs RNA Extraction B is plotted; perfect reproducibility would be along the x = y line. (0.51 MB PDF) [file pone.0010337.s004.pdf]
